# Transcriptional Down-Regulation and rRNA Cleavage in *Dictyostelium discoideum* Mitochondria during *Legionella pneumophila* Infection

**DOI:** 10.1371/journal.pone.0005706

**Published:** 2009-05-27

**Authors:** Chenyu Zhang, Adam Kuspa

**Affiliations:** 1 Verna and Marrs McLean Department of Biochemistry and Molecular Biology, Baylor College of Medicine, Houston, Texas, United States of America; 2 Department of Molecular and Human Genetics, Baylor College of Medicine, Houston Texas, United States of America; University of Liverpool, United Kingdom

## Abstract

Bacterial pathogens employ a variety of survival strategies when they invade eukaryotic cells. The amoeba *Dictyostelium discoideum* is used as a model host to study the pathogenic mechanisms that *Legionella pneumophila*, the causative agent of Legionnaire's disease, uses to kill eukaryotic cells. Here we show that the infection of *D. discoideum* by *L. pneumophila* results in a decrease in mitochondrial messenger RNAs, beginning more than 8 hours prior to detectable host cell death. These changes can be mimicked by hydrogen peroxide treatment, but not by other cytotoxic agents. The mitochondrial large subunit ribosomal RNA (LSU rRNA) is also cleaved at three specific sites during the course of infection. Two LSU rRNA fragments appear first, followed by smaller fragments produced by additional cleavage events. The initial LSU rRNA cleavage site is predicted to be on the surface of the large subunit of the mitochondrial ribosome, while two secondary sites map to the predicted interface with the small subunit. No LSU rRNA cleavage was observed after exposure of *D. discoideum* to hydrogen peroxide, or other cytotoxic chemicals that kill cells in a variety of ways. Functional *L. pneumophila* type II and type IV secretion systems are required for the cleavage, establishing a correlation between the pathogenesis of *L. pneumophila* and *D. discoideum* LSU rRNA destruction. LSU rRNA cleavage was not observed in *L. pneumophila* infections of *Acanthamoeba castellanii* or human U937 cells, suggesting that *L. pneumophila* uses distinct mechanisms to interrupt metabolism in different hosts. Thus, *L. pneumophila* infection of *D. discoideum* results in dramatic decrease of mitochondrial RNAs, and in the specific cleavage of mitochondrial rRNA. The predicted location of the cleavage sites on the mitochondrial ribosome suggests that rRNA destruction is initiated by a specific sequence of events. These findings suggest that *L. pneumophila* specifically disrupts mitochondrial protein synthesis in *D. discoideum* during the course of infection.

## Introduction

*Legionella pneumophila* is an intracellular gram-negative bacterium that causes Legionnaires' disease, a severe form of pneumonia [1], [2], [3]. The human disease is associated with inhalation of contaminated aerosols, usually emanating from air conditioning units, where amoebal species have also been found to contain the pathogen [4], [5], [6]. The bacterium attacks the human alveolar macrophage cells when inhaled. Following uptake into target cells, the bacterium occupies a phagosomal compartment that fails to acidify and to fuse with lysosome, therefore, escaping the normal degradation pathway [7], [8]. Shortly after internalization, the *L. pneumophila* containing vacuole is surrounded by host mitochondria and small vesicles derived from the endoplasmic reticulum (ER) [7], [9], [10]. As the infection progresses ER membrane surrounds the vacuole and the bacterium starts to multiply, eventually lysing the host cell. Alternatively, *L. pneumophila* can mediate its own release without lysing the host cell [11]. None of these cellular hallmarks is found in hosts that have engulfed dead bacteria or avirulent strains of the bacteria [7].

Two bacterial secretion systems have been associated with the pathogenesis of *L. pneumophila*. The bacteria use these secretion machines to transfer effector molecules, including proteins, into the host cell and also for the horizontal transfer of virulence genes between bacterial species [12]. The Dot/Icm complex, a type IV secretion apparatus, is essential for intracellular growth and pathogenesis of *L. pneumophila* [13], [14]. This secretion apparatus also promotes uptake and allows the establishment of the replication vacuole [15]. An Lsp system, a type II secretion complex, is also indispensable for pathogenesis in amoeba [16], [17].

*Dictyostelium discoideum* is a social amoeba found in the soil that has unicellular and multicellular life cycle phases [18]. Upon starvation, it develops into a fruiting body and forms spores. In nature, *D. discoideum* cells are proficient phagocytes that feed on soil bacteria. Thus, *D. discoideum* and other amoebae are likely to serve as the natural hosts for *L. pneumophila* and as such they may act as reservoirs of bacteria that pose additional risks of exposure to human populations [19], [20]. It has been demonstrated that *L. pneumophila* can infect *D. discoideum* amoebae in a manner similar to the way they infect human cells so *L. pneumophila* infection of *D. discoideum* has been suggested to be a good model for studying host-interactions with this pathogen [21], [22].

As in all eukaryotes, *D. discoideum*'s mitochondria carry their own genome that encodes RNAs and proteins for mitochondrial ribosomes as well as proteins for energy production [23]. In *D. discoideum*, disruption of the large subunit ribosomal RNA in a subpopulation of mitochondria results in defective chemotaxis and phototaxis, but not abnormal cell growth [24]. Intriguingly, physical contacts established between the nascent *L. pneumophila* containing phagosomes and host mitochondria were also observed using electron microscopy, but the potential functional relevance of this observation has not been established [9]. We report a new means by which *L. pneumophila* may disrupt *D. discoideum* cell physiology by causing a severe reduction the level of mitochondrial messenger RNAs and by destruction of the mitochondrial large subunit ribosomal RNA (LSU rRNA).

## Materials and Methods

### Cell culture

*Legionella pneumophila* bacteria were maintained on solid agar plates BCYE (buffered charcoal yeast extract). They were inoculated and grown in AYE rich media for 24 hours prior to infection [25], [26]. *Dictyostelium discoideum* wild type strain AX4 was maintained axenically, at 22°C, in shaken liquid culture (HL-5 media) or on solid SM agar plates in association with *Klebsiella aerogenes* as a food source [27].

*Acanthamoeba castellanii* (ATCC 30234) was maintained as adherent cells in PYG media in 75 cm^2^ tissue culture flasks at 25°C [28]. Human U937 cells (ATCC CRL-1593.2) were maintained as non-adherent cells in RPMI 1640 media supplemented with 2 mM L-glutamine, 10 mM HEPES, 1 mM sodium pyruvate, 4.5 g/L glucose, 10% fetal bovine serum and 50 µg/ml gentamicin, in 75-cm^2^ tissue culture flasks, at 37°C. Macrophage-like cells were induced from U937 cells by adding 10^−8^ M phorbol-12-myristate-13-acetate (PMA, Sigma). Cells were allowed to differentiate for 48 hours prior to use in infection experiments [29].

### *Legionella pneumophila* infections

Exponentially growing *D. discoideum* cells were harvested from HL-5 shaken culture (2∼4×10^6^/ml). The cells were washed twice with phosphate buffered saline (PBS). Aliquots of 10^7^ cells, were suspended in 5 ml modified HL-5 media (without addition of glucose, pH adjusted to 6.9), distributed to 60-mm culture dishes, and incubated at 26°C overnight to allow the cells to attach and adjust to the new media [21]. *L. pneumophila* cells used for infection were harvested at a post exponential OD_600_>3.0, because the cells at this growth stage have the maximum infectivity [30]. The bacteria were washed twice in PBS buffer to eliminate any soluble extracellular factors and resuspended in PBS at the concentration needed to achieve the desired multiplicity of infection (MOI). Infections were initiated by adding 100 µl of bacterial suspension into equilibrated *D. discoideum* cultures. In control experiments, green fluorescent protein expressing *L. pneumophila* cells were used to follow the uptake of the bacteria by the amoebae. At various times, amoebae were harvested and washed repeated to remove bacteria and then examined by fluorescence microscopy. At MOI = 10, >90 percent of the amoebae had at least one *L. pneumophila* inside after 4 hours, and 100 percent contained *L. pneumophila* after 12 hours.

The infections of *Acanthamoeba castellanii* and human U937 cells were conducted in a similar way. Adherent cells were tapped off the bottom of 75-cm^2^ stock culture flasks, washed in PBS, and resuspended in appropriate infection media and allowed to reattach to the bottom of 60-mm culture dishes. The infection of *A. castellanii* was assayed at 25°C or 37°C with PYG media deprived of glucose or with non-nutrient Ac buffer, as described [28]. The infection of human U937 cells was conducted at 37°C in RPMI 1640 media without antibiotics.

*D. discoideum* cell viability during *L. pneumophila* infection was assessed from triplicate samples by diluting cells from the infection media and allowing them to recover on SM agar plates inoculated with *Klebsiella aerogenes* bacteria as a food source. *D. discoideum* colonies emanating from single cells (CFU) were scored after four days of incubation at 22°C. Total RNA from infected cells was prepared for each time-point by harvesting the culture from the entire culture dish and dissolving the cell pellet in 1 ml TRIZOL reagent (Invitrogen).

### Chemical treatments

Various types of cellular stress were induced in *D. discoideum* AX4 cells that were prepared in the same way as for *L. pneumophila* infection, as described above. The chemicals (Sigma) were added directly into cell culture. Camptothecin and etoposide were applied at 100 µm; staurosporine was used at 1 µm; H_2_O_2_ was at 1 mM; G418 was used at 100 µg/ml; chloramphenicol was used at 200 µg/ml. All the treatments were carried out at 26°C, the same temperature used for *L. pneumophila* infection. Total RNA was collected at various times after the addition of the chemicals and examined for cleavage of LSU rRNA by northern blot analyses.

### Molecular analyses of LSU rRNA

The oligonucleotides used to study mitochondrial rRNAs are listed in (Table 1). The *D. discoideum* mitochondrial LSU rRNA (GenBank NC_000895) was amplified by reverse transcription polymerase chain reaction (RT-PCR) from a preparation of total RNA. In some experiments, RNA was purified from isolated mitochondria as described [31]. The antisense primer Dd3′-2863 was used to amplify the first strand cDNA, which then served as template for primers Dd5′-144 and Dd3′-2518 to amplify the entire mitochondrial LSU rRNA. The resulting product was digested with *Cla*I and *Hae*III to generate four DNA fragments used as hybridization probes that correspond to segments of the rRNA. The approximate locations of the cleavage sites were determined by northern blot analysis using these four *D. discoideum* DNA probes and were estimated from the rRNA fragment lengths and the known length of the LSU rRNA. This information was used to design primers to map the cleavage sites precisely using the rapid amplification of cDNA ends (RACE) technique. The *A. castellanii* mitochondrial LSU rRNA (GenBank NC_001637) probe was amplified from its genomic DNA using primers Ac5′-73 and Ac3′-2679 as described previously. The human mitochondrial LSU rRNA (GenBank NC_001807) probe was amplified from human genomic DNA using primers Hs5′-18 and Hs3′-1545 as described.

**Table 1:** Oligonucleotide primers used to detect and map rRNA cleavage events.

Oligonucleotide primer	Sequence (5′- 3′)
Dd3′-2863	atacgtcatccatatttagt
Dd5′-144	aagaagaaacgcagtgaagtgaaac
Dd3′-2518 (GSP1)	tagatagggaccaaactgtctcacg
Dd3′-2243 (GSP2)	gcaaacttatgccattacactcacc
Dd3′-2169 (GSP3)	tctttagaaggttaccgccccagtc
Dd3′-1391 (GSP1)	tatgcgcatcgatagtttattgtca
Dd3′-1351 (GSP2)	tcgcgtaaccctaatcagcttacct
Dd3′-1271 (GSP3)	ctcatatcagcattcttaattccga
Ac5′-73	TTTCTGGCGCCTAATCGAGC
Ac3′-2679	TACGCGTAAACCATTCGTTACCA
Hs5′-18	acccactccaccttactaccagaca
Hs3′-1545	gggtgggtgtgggtataatactaag

Dd, *Dictyostelium discoideum*; Ac, *Acanthamoeba castellanii*; Hs, *Homo sapiens*.

Antisense primers Dd3′-2243 and Dd3′-2169 were used as gene specific primers (GSP) to run the 5′ RACE, modified from the manufacturer's protocol (Invitrogen). Briefly, the first strand cDNA was amplified by GSP1 Dd3′-2518 with Superscript III reverse transcriptase (Invitrogen) at 55°C, followed by capping the 5′ end of the cDNA with dCTP using terminal deoxynucleotidyl transferase. An anchor primer with a polyG tail (Invitrogen) and a nested GSP2 Dd3′-2243 were used to PCR amplify the capped cDNA. A universal anchor primer without polyG tail (Invitrogen) and the third GSP3 Dd3′-2169 were used to run nested PCR to confirm the presence of the required cDNA species. The PCR products were TOPO cloned into TA vectors and sequenced. The rRNA primary cleavage sites and the 3′ side secondary cleavage sites were determined by aligning the cDNA sequences next to polyC tails to the original rRNA sequences. After the primary cleavage site was determined, another set of primers GSP1 Dd3′-1391, GSP2 Dd3′-1351 and GSP3 Dd3′-1271 were designed to the 5′ side of the primary cleavage site. The 5′ side secondary cleavage site was determined in a similar way that the other two cleavage sites were resolved.

The secondary structure of *D. discoideum* mitochondrial LSU rRNA was derived from comparative prediction modeling [32]. The high-resolution ribosome crystal structure of the halobacterium *Haloarcula marismortui* was used as a reference model to map *D. discoideum* mitochondrial LSU rRNA structure in three-dimensions, using PDB 1FFK coordinates [33], [34]. RasMol software was used to manipulate and render the three-dimensional structures [35].

## Results

### Mitochondrial mRNA levels decrease during *L. pneumophila*

**Figure 1:**
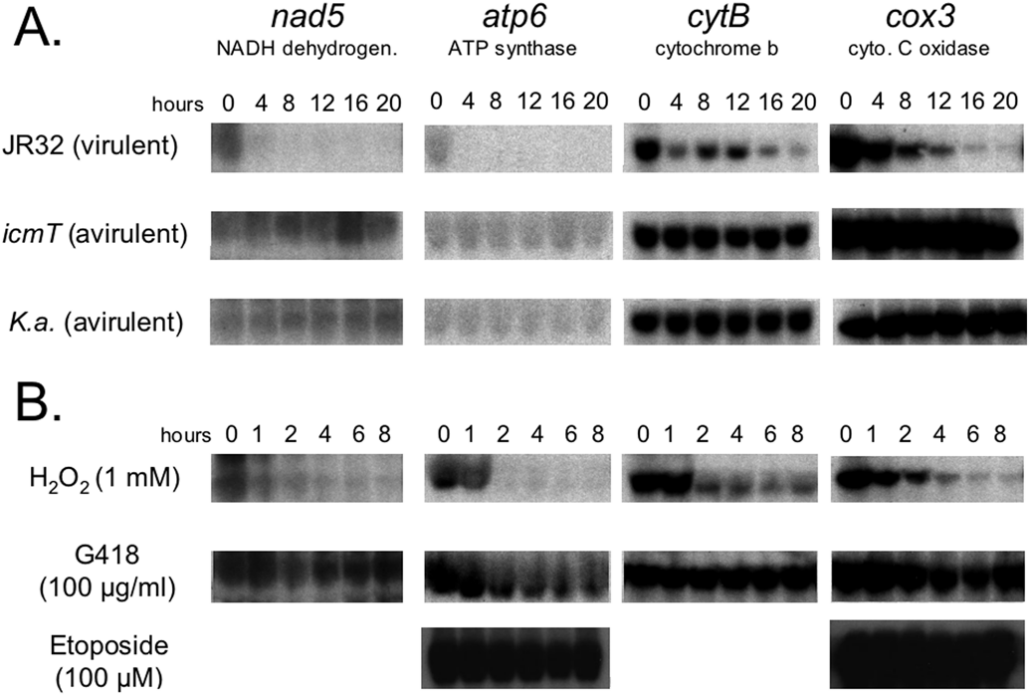
Decrease in *D. discoideum* mitochondrial mRNAs during *L. pneumophila* infection. (A) *D. discoideum* cells were infected with *L. pneumophila* (JR32) at an M.O.I. of 10 and RNA was extracted at the indicated times and analyzed on northern blots. Substantial cell death occurred after 8 hours in this experiment (data not shown). Messenger RNA for the mitochondrial-encoded genes examined displayed two modes of decline; rapid (*nad5* and *atp6*) and gradual (*cobA* and *cox3*). As controls, cells were also exposed to avirulent *L. pneumophila* (*icmT* mutant) or the food bacteria *K. aerogenes*. (B) The mRNA levels of these genes were also examined under conditions that kill *D. discoideum* cells by oxidative stress (hydrogen peroxide), inhibition of protein synthesis (G418, an aminoglycoside), inhibition of DNA synthesis (Etoposide, a topoisomerase II inhibitor).

In the course of carrying out transcriptional profiling of *D. discoideum* during infection with *L. pneumophila* we observed a decrease in the steady-state levels of a number of mRNAs that are related to mitochondrial function (data not shown). Given the reported association of *L. pneumophila*-containing phagosomes with the host cell mitochondria, we examined this finding more closely. We assessed the steady-state mRNA levels of four mitochondria-encoded genes, each of which code for one subunit of each of the four complexes of the electron transport chain [23]. These four genes; *nad5* (NADH dehydrogenase subunit), *atp6* (ATP synthase, F_0_ subunit), *cytB* (cytochrome b), and *cox3* (cytochrome c oxidase subunit) are transcribed from the mitochondrial genome as part of four distinct polycistronic primary transcripts [36]. After infection with the virulent *L. pneumophila* strain JR32, we observed two modes of mRNA decline, *nad5* and *atp6* levels decreased dramatically within the first 4 h of infection, while *cytB* and *cox3* levels declined more gradually throughout the infection (Figure 1A). Exposure of cells to an avirulent *L. pneumophila* strain (*icmT* mutant) or *Klebsiella aerogenes* (a bacterium commonly used as a laboratory food source for *D. discoideum*) had no detectable effect on transcript levels. In these experiments, cell viability began to decrease rapidly after 8 h, so the decline in *nad5* and *atp6* mRNAs appears to precede cell killing.

We next tested whether the decrease in mitochondrial transcripts could result from any form of cellular stress by treating cells with several toxins known to kill cells in different ways. The pattern of transcript decrease observed with *L. pneumophila* infection was closely reproduced by treatment with hydrogen peroxide, but not by treatment with the aminoglycoside G418, or the topoisomerase II inhibitor etoposide (Figure 1B). The oxidative stress induced by hydrogen peroxide is known to lead to mitochondrial damage, so this suggests that *L. pneumophila* infection leads specifically to a rapid disruption of mitochondrial function by inducing oxidative stress, or conditions that resemble oxidative stress.

### Cleavage of mitochondrial LSU rRNA during *L. pneumophila* infection

**Figure 2:**
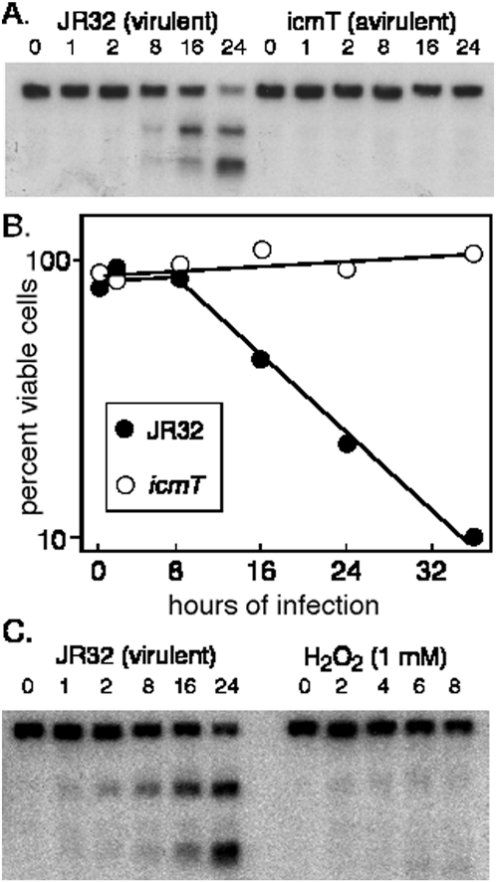
Cleavage of mitochondrial LSU rRNA during infection. (A) *D. discoideum* cells were infected with *L. pneumophila* at an MOI of 10. Total RNA was extracted after 1, 2, 8, 16 and 24 hours of infection and probed on a northern blot with a DNA fragment corresponding to the 5′ end (basepairs 144 to 377) of *D. discoideum* mitochondrial LSU rRNA. (B) The same infection was used to determine *D. discoideum* cell viability by scoring the number of viable cells by determining the number of colonies produced after recovery of the infected cells on bacterial lawns. Infection with the virulent strain JR32 (closed circles); infection with the avirulent strain *icmT* (open circles). (C.) *D. discoideum* cells were infected either infected with *L. pneumophila*, or treated with 1 mM hydrogen peroxide, as described above and mitochondrial LSU rRNA was visualized by northern blots.

*D. discoideum* mitochondrial LSU rRNA is typically used as a normalization control in northern blots, but when we probed for the LSU rRNA it appeared to be cleaved during *L. pneumophila* infection. Using the LSU rRNA 5′ end as probe, smaller fragments appeared in RNA samples isolated from infected *D. discoideum* cultures, and these fragments accumulated as the infection progressed as judged by the increase in band intensities (Figure 2A). A larger fragment appeared to accumulate earlier in the course of infection than the smaller fragments, suggesting a specific and sequential cleavage, with a primary cleavage followed by secondary cleavage events. The cleavage did not occur when cells were exposed to avirulent strains *L. pneumophila* (Figure 2A; and see below). We also demonstrated by northern blot analyses that cytoplasmic rRNAs remained intact during infection by probing the same RNA samples that contained cleaved mitochondrial LSU rRNA (data not shown). The cleavage of the mitochondrial LSU rRNA also correlated with decreased survival of *D. discoideum* during infection (Figure 2B). The presence of detectable cleavage at 8 hours post infection, before any detectable loss in cell viability, suggests that LSU rRNA cleavage precedes cell killing. Contrary to the similar effect that virulent *L. pneumophila* and hydrogen peroxide had on mitochondrial mRNA levels, hydrogen peroxide treatment did not induced LSU rRNA cleavage in cells (Figure 2C).

### LSU rRNA cleavage is not caused by other forms of cytotoxic stress

It has been observed that virulent *L. pneumophila* infection induces host cell apoptosis in mammalian cells [37], [38], [39]. To begin to test whether the observed cleavage of mitochondrial LSU rRNA is a host cell response to any stress, or caused by *L. pneumophila* directly, we induced stress in *D. discoideum* cells by a variety of chemical treatments and assessed mitochondrial rRNA integrity on northern blots. Camptothecin and etoposide are apoptosis inducers that poison eukaryotic DNA topoisomerases I and II, respectively [40], [41]. Staurosporine is a potent protein kinase C inhibitor [42] while the antibiotics G418 and chloramphenicol inhibit eukaryotic cytoplasmic and mitochondrial ribosomal protein synthesis, respectively, and hydrogen peroxide induces oxidative stress in cells. These treatments caused *D. discoideum* cell death but none of them led to the mitochondrial LSU rRNA cleavage observed during *L. pneumophila* infection (Table 2). These results suggest that the observed rRNA cleavage is caused by *L. pneumophila* infection rather than a general host cell stress response.

**Table 2:** Chemical-induced stress and mitochondrial rRNA integrity.

Chemical reagent	Expected Target	Concentra-tion used^1^	LSU rRNA cleaveage^2^
Camptochecin	DNA topoisomerase I	100 µM	Not detected
Etoposide	DNA topoisomerase II	100 µM	Not detected
Staurosporine	PKC inhibitor	1 µM	Not detected
H_2_O_2_	Oxidative stress	1 mM	Not detected
G418	Cytoplasmic ribosome	100 µg/ml	Not detected
Chloramphenicol	Mitochondrial ribosome	200 µg/ml	Not detected

1 Treatments were for 20 hours prior to isolation of total RNA.

2 Integrity of the LSU rRNA was determined by northern blot analyses as described in Figure 2.

### LSU rRNA cleavage requires both the type II (lsp) and type IV (dot/icm) secretion systems

All of the *L. pneumophila* pathogenic strains that have been sequenced so far carry type IV Dot/Icm and type II Lsp secretion systems that are believed to be required for pathogenesis [43], [44]. Most of the bacteria's pathogenic functions reported so far require the integrity of one or the other of these secretion systems [14], [16], [17], [45], [46]. We tested which of these two *L. pneumophila* secretion pathways are required for the rRNA cleavage to take place. The *icmT* and *dotA* genes reside within two different gene clusters that encode the core components of the type IV secretion machinery [13], [47], [48]. The *lspF* and *lspG* genes are clustered together and encode the core of the type II machinery, while the *lspDE* genes encode another component of the type II secretion machinery [43]. The type IV mutants, *icmT* and *dotA*, as well as the type II mutants *lspF*, *lspG* and *lspDE* were tested for their ability to induce rRNA cleavage. None of these mutants were observed to induce cleavage of the mitochondrial rRNA after mixing them with *D. discoideum* (Table 3). These results indicate that both the type IV and the type II secretion systems must be functional for *D. discoideum* mitochondrial LSU rRNA cleavage to occur.

**Table 3:** Effect of *L. pneumophila* type II and type IV secretion mutants on *D. discoideum* mitochondrial rRNA cleavage.

Strain	Phenotype	Source	Human toxicity	Amoebae toxicity	Cleave rRNA
JR32	Wild type	[47]	Yes	Yes	Yes
icmT	Type IV	[47]	No	No	No
LP02/(*thy* ^−^ *dot* ^+^)	Wild type	[14]	Yes	Yes	Yes
LP03/(*thy* ^−^ *dotA* ^−^)	Type IV	[14]	No	No	No
GL84/(*thy* ^−^ *dotA* ^−^) pDOT1(*thy* ^+^ *dotA* ^+^)	Type IV rescue	Isberg, R.	Yes	Yes	Yes
130b	Wild type	Cianciotto, N.	Yes	Yes	Yes
NU275/*lspF*	Type II	[45]	Yes	No	No
NU259/*lspG*	Type II	[17]	Yes	No	No
NU258/*lspDE*	Type II	[17]	Yes	No	No

*D. discoideum* AX4 cells were infected by various type IV or type II secretion mutants of *L. pneumophila* and their parental strains. The human toxicity data are from the cited references. The *D. discoideum* cell viability was determined by plating for viable cells in an assay for colony forming unit and mitochondrial LSU rRNA integrity was assessed by northern blot analyses as described in Figure 2 (data not shown).

We also tested the pathogenicity of these type IV and type II mutants on *D. discoideum*. As expected, none of these *L. pneumophila* mutants killed *D. discoideum*, as their parental wild type strains did, which corroborates the findings using other amoebae as hosts (Table 3). Since we could have observed some LSU rRNA cleavage without death of the *Dictyostelium* cells, but did not, a strict correlation exists between the pathogenesis of *L. pneumophila* and the cleavage of *D. discoideum*'s mitochondrial LSU rRNA.

We attempted to identify possible *L. pneumophila* enzymes responsible for the rRNA cleavage by using a candidate gene approach. We inspected all predicted ribonuclease genes in the genomic sequence of *L. pneumophila* and excluded genes predicted to encode ribonucleases with defined functions (10). We found a single predicted T2 ribonuclease with an RNase core domain plus uncharacterized domains that we hypothesized might be involved in determining substrate specificity (GenBank AE017354). A deletion mutant of the gene encoding this protein, provided by the laboratory of Ralph Isberg, was assayed for LSU rRNA cleavage activity upon infection of *D. discoideum*. Mitochondrial LSU rRNA cleavage was observed, just as in wild type *L. pneumophila* infection, indicating that this protein is not responsible for the rRNA cleavage activity (data not shown).

### Determination of the LSU rRNA cleavage sites

**Figure 3:**
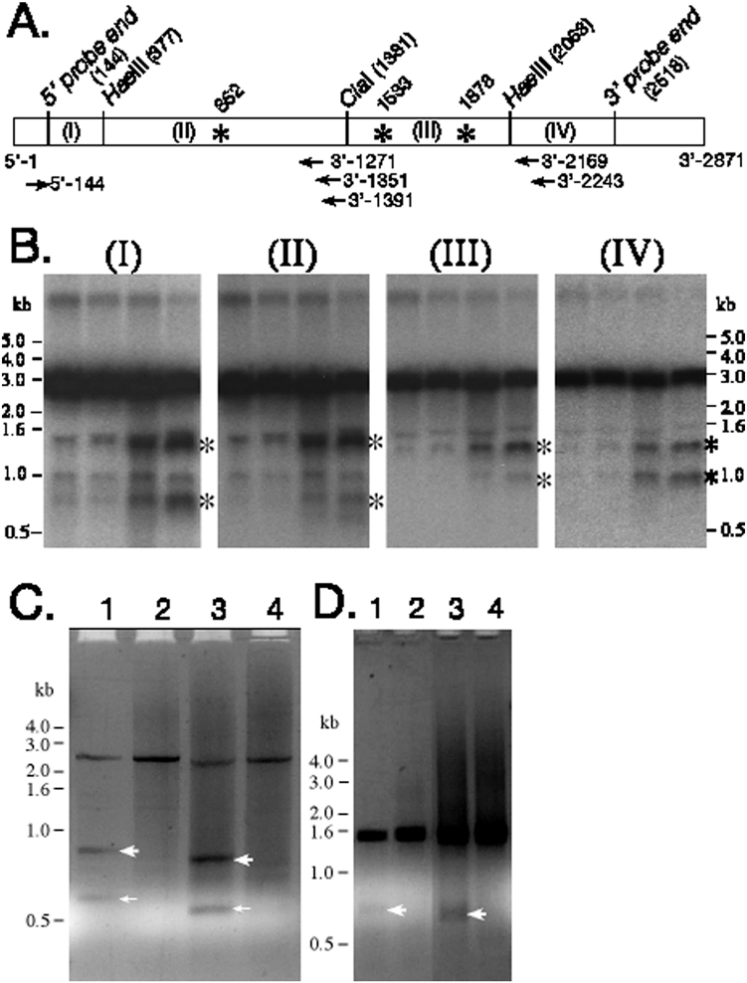
Determining the sites of LSU rRNA cleavage. (A) A map of *D. discoideum* mitochondrial LSU rRNA showing the position of restriction sites and oligonucleotide primers (arrows) used to define the cleavage sites (asterisks). LSU rRNA was amplified by RT-PCR and digested with *Cla*I and *Hae*III into four segments (I, II, III and IV). (B) The fragments in A were used individually as probes and hybridized to northern blots of total RNA extracts from *D. discoideum* infections. Labeled bands were used to infer the approximate cleavage sites on the rRNA. For example, the major cleavage site is ∼1.5 kb, and one minor cleavage site is 0.8 kb, from the 5′ end of the rRNA (panels I and II, asterisks) whereas the major cleavage sites is ∼1.3 kb and the other minor cleavage site is ∼1.0 kb from the 3′-end of the rRNA (panels III and IV, asterisks). (C) The 5′RACE products from the cleaved *D. discoideum* mitochondrial LSU rRNA. Total RNA samples from *D. discoideum* infected by virulent strain JR32 (lanes 1 and 3) or infected by avirulent strain *icmT* (lanes 2 and 4) were used in RACE reactions with PCR primers 3′-2243 (lanes 1 and 2), or 3′-2169 (lanes 3 and 4), and the RACE AAP primer (see Materials and Methods). The products resulting from the major cleavage site at base 1533 (thick arrows) and the minor cleavage site at base 1878 (thin arrows) are evident on the ethidium bromide stained agarose gel (negative image). The four smaller bands were cloned and sequenced to determine the cleavage sites. (D) Determination of the 5′ side minor cleavage site by RACE. Total RNA from 20-hour virulent (JR32) infection (lane 1) and 20-hour avirulent *icmT* infection (lane 2) were used in 5′-RACE reactions with primers 3′-1351 and the RACE AAP primer. Nested PCR reactions using the products shown in lanes 1 and 2 as substrates, with primer 3′-1271 and the RACE AUAP primer (lanes 3 and 4, respectively). The lower bands in lanes 1 and 3 (arrows) were cloned and sequenced to determine the minor cleavage site at base 862.

We used RACE (Rapid Amplification of cDNA Ends) to map the specific cleavage sites on the rRNA [49]. In order to design the RACE primers we mapped the approximate locations of the cleavage sites by northern blot analyses using segments of RT-PCR-amplified mitochondrial LSU rRNA as probes (Figure 3A and 3B). Two sets of primers were designed for the RACE reactions that bordered the approximate locations of the cleavage sites. We carried out RACE reactions on samples of total RNA collected from cells that had been infected with virulent and avirulent *L. pneumophila*. Using two different RACE primers two fragments and the full-length copy of the rRNA were easily detectable, but only from cells infected with virulent *L. pneumophila* (Figure 3C). The RACE products representing sub-fragments of the copied and amplified rRNA were cloned into plasmids and the cleavage sites were then determined by sequencing the inserts (Figure 3C). The sequencing of the RACE products revealed the precise locations of one major and one minor cleavage site on the *D. discoideum* mitochondrial LSU rRNA (Figure 3A). With the major cleavage site mapped, RACE primers were designed that lay to the 5-prime side of that site and used to amplify the other minor cleavage fragment (Figure 3D).

**Figure 4:**
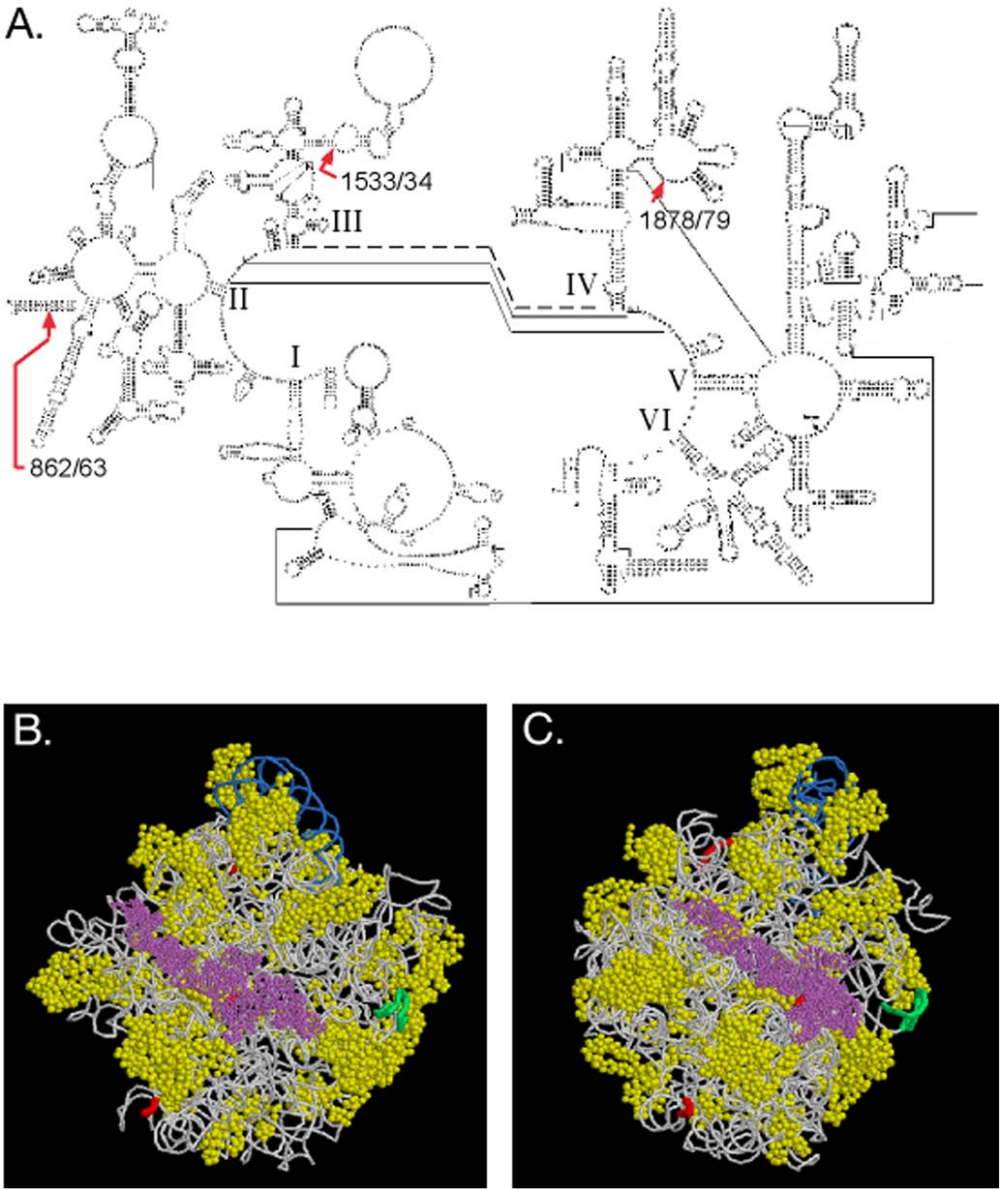
Location of the cleavage sites on the *D. discoideum* mitochondrial LSU rRNA structure. (A) Predicted secondary structure of *D. discoideum* mitochondrial LSU rRNA showing the locations of the cleavage sites (red arrows). For clarity, the rRNA is split between domains III and IV and the thick dashed line indicates the covalent linkage between the two halves. Lines indicate predicted basepairs in the three dimensional structure between bases that widely separated in the primary sequence. The domains are labeled in roman numerals near where their secondary structure emanates from the center. (B) LSU rRNA cleavage sites (red) mapped onto the three-dimensional structure of the *Haloarcula marismortui* mitochondrial ribosome (pdb 1ffk). The LSU rRNA (grey line), the 5S rRNA (blue line) and ribosomal proteins (yellow) are shown. The rRNA sarcin/ricin loop is indicated (green). The points of contact with the small subunit (purple) in domain IV of the rRNA secondary structure are where essential ribosomal functions are carried out and where the cleavage site at base 1878 is located (red). (C) Predicted structure in B rotated 60 degrees counterclockwise on the vertical axis.

The northern blot data indicates that the LSU rRNA is cleaved into discrete fragments and the unambiguous ends identified by sequencing each of the RACE products strongly suggests that the fragmentation results from cleavage at discrete sites. The major cleavage site mapped between nucleotides 1533 and 1534 (Figure 3A). A minor cleavage site on the 5′ side of the major site mapped between nucleotides 862 and 863 and another minor cleavage site on the 3′ side of the major site mapped between nucleotides 1878 and 1879. Because the eukaryotic mitochondrial ribosomes are thought to have shared a common ancestor with archaebacterial ribosomes, we mapped the cleavage sites onto the predicted secondary structure of the *D. discoideum* mitochondrial LSU rRNA based on the halobacteria *Haloarcula marismortui* rRNA structure [33], [50] (Figure 4A). When mapped onto the corresponding regions of the *H. marismortui* rRNA high-resolution crystal structure, the major cleavage site is predicted to be on the surface of the ribosome (Figure 4B). This is consistent with the expectation that the initial cleavage site is accessible on the intact ribosome similar to the sarcin/ricin loop, a distinct site that is subject to cleavage by those toxins (Figure 4B and 4C). The minor cleavage site at base 1878 is predicted to be in domain IV of the LSU rRNA (Figure 4A), where essential functional contacts are made between the ribosome's large and small subunits (Figure 4B and 4C). The minor cleavage site at base 862 and is predicted to be deep within the ribosomal structure (Figure 4C).

### No detectable rRNA cleavage in *Acanthamoeba castellanii* or human U937 cells

**Figure 5:**
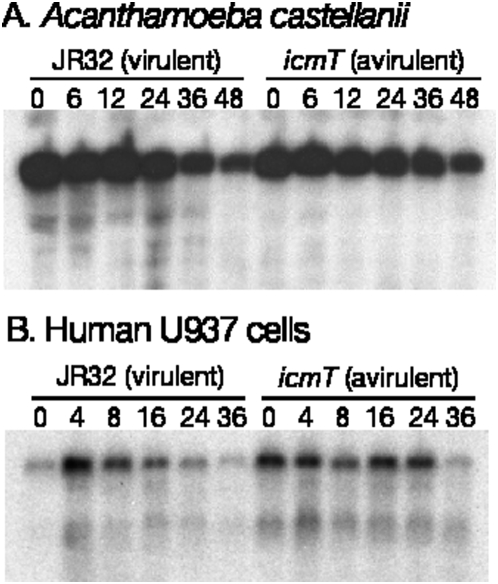
No LSU rRNA cleavage in Acanthamoeba or human cells during *L. pneumophila* infection. (A) *A. castellanii* (ATCC 30234) were infected by *L. pneumophila* in Ac buffer at 37°C. (B) Human U937 cells (ATCC CRL-1593.2) were infected by *L. pneumophila*. Total RNA samples were probed on northern blots with a *A. castellanii* or human mitochondrial LSU rRNA probes. In each experiment, the infection was confirmed by monitoring the internalization of GFP-labeled bacteria and host cell killing (data not shown). Experiments were carried out three times and a representative experiment is shown.

We tested whether LSU rRNA cleavage occurs in other cells infected with *L. pneumophila*. *Acanthamoeba castellanii* and human U937 cells are two widely used models of *L. pneumophila* infection [28], [29]. We infected these cells using established protocols and assayed for host cell viability and mitochondrial LSU rRNA integrity, but we did not observe any cleavage events during either of these infections (Figure 5). This result with human cells is not surprising since the entire structural domain where the primary cleavage site is located in *D. discoideum* LSU rRNA has been lost in human LSU rRNA [32], [51]. However, in *A. castellanii*, the major cleavage site and the minor site at base 1878 are conserved, whereas the minor cleavage site at base 832 is within a loop that is unique in *D. discoideum* [32].

## Discussion

We have described major changes in the stability of *D. discoideum*'s mitochondrial RNAs during *L. pneumophila* infection. The LSU rRNAs of the mitochondrial ribosomes are cleaved at specific sites and there is a decrease in the steady-state level of each of the mitochondrial mRNAs that we examined. Some mRNA levels drop to undetectable levels within four hours of the start of the infection while other mRNAs decrease more gradually. Thus, some mRNA levels decrease well before cell death is detectable after 8 hours. We found that we could reproduce this pattern of mRNA loss by treatment with hydrogen peroxide, but not by treatments with other toxic agents. The production of hydrogen peroxide and other reactive oxygen species (ROS) by the mitochondria and the protective responses to ROS toxicity are well described [52]. The incomplete reduction of oxygen by the electron transport chain results in ROS and this causes reduction in the flux through the tricarboxylic acid cycle through the reversible unfolding of redox-sensitive enzymes such as aconitase [53] and, eventually, loss of mitochondrial mRNAs [54]. Thus, *L. pneumophila* infection appears to induce an effect on host cell mitochondria reminiscent of an oxidative stress response.

Our results suggest that infection results in a complete shut down of mitochondrial gene expression. Although we did not test the expression of every gene encoded by the mitochondrial genome (mtDNA), the four genes that we did test are transcribed initially as part of four polycistronic transcripts that comprise about half of the entire coding capacity of the mtDNA [36]. If the decrease in mRNAs that we observed resulted from transcriptional initiation, it is reasonable to assume that the mRNA encoding all of the proteins that come from those transcripts decrease during infection. Farbrother, et al., recently reported alterations in the transcriptome that occur during *L. pneumophila* infection under similar conditions [55]. Using cDNA hybridization to oligonucleotide microarrays, they reported that the mRNA for genes such as *cox3* increase slightly during progression of the infection, whereas our northern analyses clearly indicate that *cox3* mRNA decreases quite dramatically. We cannot account for this discrepancy.

In addition to the effect on mRNAs, we also found that *L. pneumophila* infection results in cleavage of the mitochondrial LSU rRNA, an effect that could not be mimicked by treatment with hydrogen peroxide or any other cytotxic agents that we tested. Hydrogen peroxide has been reported to cause mitochondrial rRNA destruction in other systems but we saw no evidence of this in *Dictyostelium* [56]. If the specific cleavage and destruction of LSU rRNA were an intrinsic response of *D. discoideum* to particular forms of stress, we would have expected to observe it under different conditions. We tested six different chemical agents that induce stress by different means and eventually kill cells, but we never observed LSU rRNA cleavage (Table 2). Thus, we favor the notion that *L. pneumophila* induces the cleavage directly, either by secretion of a ricin-like RNase or by specific alteration a host cell enzyme.

The identification of the protein(s) responsible for the LSU rRNA cleavage, or the selection of a *D. discoideum* mutant fully resistant to the rRNA cleavage during infection, are required to further characterize the rRNA cleavage mechanism and its relationship to *L. pneumophila* pathogenesis. We demonstrated a requirement for both the type II and the type IV secretion systems of *L. pneumophila* in mitochondrial LSU rRNA cleavage. The simplest interpretation of this result is that the protein(s) responsible for cleavage are bacterial cargo of the type II or the type IV systems. However, since both secretion systems are required for productive *L. pneumophila* infection, it is equally plausible that the progression of cellular pathogenesis is required to set up the proper environment for rRNA cleavage, independent of whether or not the cleavage is carried out by a bacterial enzyme.

We attempted to identify the *L. pneumophila* protein responsible for the rRNA cleavage using a candidate gene approach. We examined all of the predicted protein products of the *L. pneumophila* genome for those containing any known RNase or ricin/sarcin domains [43]. The best candidate that we identified is a T2 RNase family enzyme that contains additional amino acid sequences that are not found in other “housekeeping” ribonucleases. An *L. pneumophila* deletion mutant in this enzyme is still capable of inducing LSU rRNA cleavage in *D. discoideum*. Considering that the presumed initial cleavage site on the surface of the ribosome has not been previously recognized as a site of attack by ribonuclease toxins, it is possible that a new family of rRNA nucleases carries out the rRNA cleavage.

The sequential appearance of the LSU rRNA fragments during *L. pneumophila* infection is suggestive of a regulated destruction of the mitochondrial ribosomes. We mapped the locations of the cleavage events onto the predicted structure of the *D. discoideum* LSU rRNA based on high-resolution structures of archaebacterial ribosomes. The first cleavage event is predicted to occur on the surface of the ribosome while two secondary events are predicted to occur within the ribosomal structure. We can hypothesize two scenarios that would produce the observed order of cleavage. The cleavage of the primary site may destabilize the overall structure of the ribosome during translation, causing the small subunit to dissociate and thus exposing the two secondary sites for cleavage. Alternatively, cleavage of the primary site could occur at any time in the translation cycle, rendering the large subunit incapable of engaging in translation or associating with the small subunit, leaving exposed the two sites that are normally rendered inaccessible by interactions with the small subunit. Independent of the mechanism of cleavage, it is likely that cleavage of the rRNA at the sites buried in regions essential to translation would abolish the normal function of the mitochondrial ribosome.

Although the mitochondrial LSU rRNA cleavage is observed in *D. discoideum* but not in *A. castellanii* or human host cells, it does not rule out the possible attack by *L. pneumophila* on host cell mitochondria in other organisms. One intriguing question remains as to the molecular route that is exploited to attack the host cells' mitochondria. Are physical contacts between the pathogen-containing phagosome and host cell mitochondria required, or do *L. pneumophila* proteins secreted into the cytoplasm exploit the normal mitochondrial import machinery of the host? We tested for rRNA cleavage in *D. discoideum cluA* mutants, which have a phenotype of abnormal distribution of tightly clustered mitochondria [57]. The clustered mitochondria would be expected to preclude interactions with other organelles because the mitochondria are in close apposition with each other in this mutant. Somewhat surprisingly, we detected a similar level of rRNA cleavage in infected *cluA* mutant cells and in wild-type cells (unpublished observations). This suggests that the possible bacterial proteins responsible for the cleavage need not be delivered to the host cell mitochondria through direct association with the pathogen-containing phagosome, or replicative vacuole.

We have established a correlation between LSU rRNA cleavage and host cell killing with the available mutants. The *L. pneumophila* strains that kill *D. discoideum* also cleave their rRNA, while none of the avirulent *L. pneumophila* strains cleave the rRNA. However, as discussed above, this correlative relationship does not warrant causality between mitochondrial rRNA cleavage and the pathogenesis of *L. pneumophila* infection in *D. discoideum*. The establishment of such a causal relationship could be based on the identification of proteins responsible for the cleavage, or the availability of a *D. discoideum* mutant that is resistant to the cleavage. It also should be pointed out that while the type IV apparatus is required for the pathogenesis in both animal cells and amoebae, while the type II secretion system is dispensable for the virulence in humans. This supports the notion that different pathogenic mechanisms operate during *L. pneumophila* infection of mammalian cells and amoebae.

Early interactions with the host cell are essential to the establishment of a specialized *L. pneumophila* containing phagosome and to the evasion of phagosome-lysosome fusion that would otherwise lead to degradation of the bacteria. Horwitz et al. reported an association between *L. pneumophila* containing phagosomes and host cell mitochondria soon after the internalization of the bacteria, which were extended by the demonstration of physical contacts between the two compartments [9], [58]. Although these observations have not been further explored as a potential pathogenic mechanism, we have now shown that *L. pneumophila* infection of *D. discoideum* results in the destruction of the mitochondrial large subunit ribosomal RNA (LSU rRNA). This suggests that the host cell mitochondria are a target of modulation by *L. pneumophila* during infection. Since the destruction of the mitochondrial rRNA correlates temporally with host cell death, we are now in a position to explore how compromised mitochondrial protein synthesis might contribute to a productive *L. pneumophila* infection and the demise of the host cell.
